# Primary Care Quality and Revisit Intention Among Hong Kong Older Adults: A Cross-Sectional Study

**DOI:** 10.3390/healthcare14183054

**Published:** 2026-09-17

**Authors:** Wang-Kin Chiu, Mei Mei Lau, Tommy K. C. Ng, Ben Y. F. Fong, Winston W. C. Tse, Vincent T. S. Law, Sammantha S. P. Ho, Zhanming Liang, William C. W. Wong

**Affiliations:** 1 Division of Science, Engineering and Health Studies, School of Professional Education and Executive Development, The Hong Kong Polytechnic University, Hong Kong, China; tommy.ng@cpce-polyu.edu.hk (T.K.C.N.); ben.fong@cpce-polyu.edu.hk (B.Y.F.F.); 2 Division of Business and Hospitality Management, School of Professional Education and Executive Development, The Hong Kong Polytechnic University, Hong Kong, China; may.lau@cpce-polyu.edu.hk; 3 Faculty of Health and Social Sciences, The Hong Kong Polytechnic University, Hong Kong, China; 4 Division of Social Sciences, Humanities and Design, School of Professional Education and Executive Development, The Hong Kong Polytechnic University, Hong Kong, China; vincent.law@cpce-polyu.edu.hk; 5 Hong Kong Institute of Asia-Pacific Studies, The Chinese University of Hong Kong, Hong Kong, China; spsho@cuhk.edu.hk; 6 College of Business, Law and Governance, James Cook University, Townsville 4811, Australia; zhanming.liang@jcu.edu.au; 7 Department of Family Medicine and Primary Care, The University of Hong Kong, Hong Kong, China; wongwcw@hku.hk; 8 Department of General Practice, The University of Hong Kong-Shenzhen Hospital, Shenzhen 518053, China

**Keywords:** Primary Care Assessment Tool (PCAT), first contact accessibility, first contact utilisation, ongoing care, coordination, comprehensiveness, family-centeredness, community orientation, perceived service quality, satisfaction, trust, revisit intention, primary healthcare

## Abstract

**Background:** The healthcare system of Hong Kong is facing a growing burden arising from chronic diseases and patient demands because of the rapid ageing and healthcare workforce shortage. Primary care is widely recognised as the most cost-effective way in improving health of the people. This study aimed to understand how older adults’ experiences of primary care in Hong Kong may impact the service utilisation and revisit intention of primary care services. **Methods:** This correlational study used a combination of cluster, purposive, and snowball sampling methods and collected 573 valid responses from Hong Kong residents aged 60 years or above. A modified version of the Primary Care Assessment Tool Chinese Version (PCAT-C), requiring an estimated 15 min to complete, was used to measure the perceived service quality, satisfaction, trust, and revisit intention. Data collections were performed between March 2025 and November 2025. A partial least squares structural equation modelling (PLS-SEM) was performed in the statistical software SmartPLS 4.0 to test the relationships of various variables. **Results:** By analysing the 573 valid samples (*M*_age_: 74.2, *SD*: 7.3; female: 80.5%) from the 607 total collected responses, ongoing care was revealed as the most influential PCAT factor in both older adults’ perceived service quality (*β* = 0.368; *p* < 0.0001) and satisfaction *(β* = 0.189; *p* < 0.0001), while trust (*β* = 0.265; *p* < 0.0001) and perceived service quality (*β* = 0.195, *p* = 0.001) were found to have positively contributed to older adults’ intention to revisit primary care services among the respective relationships among perceived service quality, satisfaction, and trust towards revisit intention. **Conclusions:** To enhance the utilisation of primary care among older adults, it is vital to improve their trust, perceived service quality, and satisfaction with primary care providers. One promising way to achieve this goal is to improve ongoing quality of care and people-centred care at the primary care level.

## 1. Introduction

Population ageing in Hong Kong is among the highest in the world, and the number of older adults is expected to increase from 1.5 million (20% of the total population) in 2021 to 2.52 million (31% of the total population) in 2039 [1]. Likewise, there has also been an increase in chronic diseases, affecting 2.2 million patients in 2021, of which 47% are older adults. The figure is expected to exceed 3 million in the next ten years. The trends of an ageing population and chronic diseases will place a significant strain on the healthcare system, especially for public hospitals. Despite older adults having only constituted 18% of the population in 2019, they accounted for over one-third of the Family Medicine Clinics and specialist outpatient clinic visits, and around half of the total number of inpatients and admissions to the Accident and Emergency [1]. The challenges have signalled the importance of reforming the existing healthcare system, by strengthening primary care, as it is considered the most cost-effective way to deliver healthcare services [2].

According to the World Health Organization (WHO), global life expectancy has increased by over eight years, rising from 64.9 years in 1995 to 73.3 years in 2024. With this upward trend, the global population aged 60 or older is projected to reach 1.4 billion by 2030, up from 1.1 billion in 2023. This ageing trend first appeared in high-income countries, but it is now becoming more pronounced in the middle-income and low-income countries [3]. For instance, for high-income countries like France, projections indicated that the population aged 65 or above will increase from 15.3 million, 22% of the total population in 2026, to 18.6 million, 27% of the total population, in 2040 [4]. For middle-income countries like Thailand, the share of the population aged 60 or older is expected to grow from 22% of the total population in 2024 to 36% by 2050 [5]. In the case of China, an upper-middle-income country, its elderly population is estimated to increase from 254 million in 2019 to 402 million in 2040 [6]. Nonetheless, as one of the low-income countries, Malawi has already experienced a huge increase in its ageing population, and its population aged over 60 will grow by 44% from 1987 to 2008, and is expected to reach 3.3 million by 2050 [7].

Globally, nations ranging from the United Kingdom to Brazil have adopted primary care as a key strategy in managing the complexities of an ageing demographic [8]. Research has shown the enormous benefits of having clinicians who have received family medicine training at the postgraduate level in the primary care team [9]. Primary care is an indispensable foundation for achieving universal health coverage and plays an essential role in forming trust relationships in the community. To meet the healthcare challenges of the 2030s, a new primary healthcare model should be developed to ensure balanced emphasis on all core functions within the primary care system [9].

In Hong Kong, where a dual-track healthcare system of public and private sectors is adopted, over 70% of primary care services are provided by the private sector, mainly the general practitioners (GP) in private clinics [10]. The Hong Kong Government has prioritised a shift in policy focus towards the primary level of care and released The Primary Healthcare Blueprint in December 2022 to outline a vision of improving the overall health of citizens and building a sustainable healthcare system. The government plans to reform the healthcare system by prioritising family-centeredness, community orientation, and prevention-oriented strategies, with early detection of diseases and timely intervention. District Health Centres or Expresses have been established in all 18 districts since 2019. They act as resource hubs and provide different aspects of care including prevention and screening of disease, management of chronic diseases, and rehabilitation services [11].

### 1.1. Tools for Assessing Strength of Primary Care

To remedy the current problems such as “doctor-shopping” behaviours, it is crucial to benchmark current service quality from the primary care users’ perspective. Several tools are available to assess the strength of primary care, including the Primary Care Assessment Tool (PCAT), Quality and Outcomes Framework (QOF), Primary Health Care Performance Initiative (PHCPI), European Primary Care Monitor Framework (EPCM) and Person-Centered Primary Care Measure (PCPCM). While all of them enable the measurement of primary care strength, there are different focuses.

The QOF, as a scheme to improve the quality of doctors, provides a variety of organisational and clinical indicators for doctors to follow. However, the QOF does not include direct measures of key primary care domains, such as first contact accessibility, ongoing care, coordination, comprehensiveness, patient-centeredness, or community orientation. The PHCPI and EPCM both cover the equity, efficiency, and economic aspects of primary care, but they neglect measures of the dimensions of family or patient-centeredness and community orientation. The PCPCM focuses more on the relationship between the primary care providers and patients although it also measures six of the seven primary care domains included in the PCAT. Moreover, as the PCPCM only has 11 items in total [12], its comprehensiveness in measuring each of the primary care domains is limited.

The PCAT is a tool to measure the quality of primary care and it has been widely used in different countries, including Brazil, Canada, China and Spain [13]. As this study aimed to gain a comprehensive understanding of primary care quality from primary care users’ perspective, the PCAT was considered the most suitable instrument. The respective version used in the current study was the modified version of the Chinese Primary Care Tool (PCAT-C), which is a translated and adapted version of the PCAT, with its items modified to reflect the specific circumstances of the Chinese population.

#### 1.1.1. Perceived Service Quality, Satisfaction, Trust, and Revisit Intention

Perceived service quality refers to patients’ awareness and perception of the quality of the healthcare service they have received [14]. It has been divided into two domains: process quality and outcome quality. The process quality is related to how healthcare service is being provided to the patients by healthcare providers. The outcome quality means how precisely the healthcare procedures and medical assessments are performed [15]. Whether the patient is satisfied is based on how the outcome of the service received by the patients meets their expectations [14]. Although patients’ satisfaction with healthcare can be attributed to many factors, scholars have highlighted some influential external factors, including age, education level, perceived health status, queuing time, and the quality of communication with healthcare providers [16].

The convergence of the abovementioned elements affects patients’ trust. Trust is an internal, psychological factor, and it is commonly described as the belief that others will do no harm and keep a promise. It has also been regarded as ‘lowering one’s guard’ and trusting something precious to other people, including one’s health. Trust can be divided into three types, including institutional trust, particular trust, and generalised trust. Institutional trust is the trust towards institutions, for instance, the healthcare system or mass media. Particular trust is the trust towards individuals, including one’s GP or family members. General trust is the trust towards other people, for example, the general public and strangers [17]. Therefore, the trust discussed in the current paper is the particular trust towards GPs. Revisit intention can simply be regarded as the action of patronisation. It is the motive or thoughts of patients in choosing the same doctor or clinic. Moreover, the willingness to continue a long-term relationship with the healthcare provider is also part of revisit intention [18]. Solely in the healthcare context, service quality and physical evidence are found to be essential factors behind patients’ revisit intention [19]. Among the elements of service quality, the responsiveness, empathy and assurance of frontline healthcare providers are often regarded as the most important ones. The physical evidence refers to tangible elements that includes waiting area comfort, cleanliness, and facility design [19]. In a systematic review of revisit intention in tourism and hospitality studies, besides service quality, factors including perceived trust, perceived destination, and experience were found positively associated with revisit intention. However, the same study revealed that variables such as accessibility, attitude, electronic word of mouth and subject norm have not been studied sufficiently regarding the correlation with revisit intention [20].

While intention is among the most important predictors for a behaviour to be triggered, as suggested by theories widely used in healthcare research such as the theory of planned behaviour [21,22,23], there are various factors at the individual and organisational levels associated with the correlation of intentions and behaviours. Some studies have reported the existence of an intention–behaviour gap, where a health behaviour does not necessarily happen after having a health behaviour intention [24,25]. Some studies use the health action process approach to explain this gap, since in the relationship of an intention and a behaviour, action planning appears a mediator [26]. A recent study has further suggested that the formation of an intention–behaviour gap should be considered in four dimensions, including the person dimension, behavioural dimension, temporal dimension, and contextual dimension. Individual differences in forming the intention, and the nature of the heath behaviour (how continuous or complex the behaviour is) should be taken into account. The nature of the gap may appear or disappear over time, and the fact that situational contexts and environmental contexts matter should also be emphasised [27].

#### 1.1.2. Relationship Among PCAT Domains, Perceived Service Quality, Satisfaction, Trust, and Revisit Intention

PCAT constructs might be related to patients’ perceived service quality and satisfaction. A study at a local community healthcare centre in China has suggested that perceived service quality by patients could be enhanced through improving different aspects in the PCAT scale [28]. Another study has noted the relationship between the usual source of care (a domain of ongoing care) and perceived service quality [29]. Care coordination has also been found to affect the quality of care in home care [30]. Moreover, PCAT constructs have been found to be positively associated with a patient’s satisfaction. In the same study, first contact accessibility, ongoing care, and family-centeredness were also suggested to be related to a patient’s revisit intention to the same primary care provider [31]. Well-delivered first contact accessibility and coordination in primary care have also been shown as indicators of higher patient satisfaction [32]. Furthermore, the relationship between a patient’s perception of service quality and satisfaction with healthcare has been demonstrated worldwide [33,34,35], and research has further suggested assurance as the most influential element in this relationship, followed by reliability and responsiveness, while empathy does not contribute significantly [35]. It has been suggested that perceived service quality is remarkably associated with trust [36,37,38], and some studies have explained this correlation by the mediating role of satisfaction [37,38]. Another study has claimed the positive association between perceived service quality and trust appears from good interaction service quality and outcome service quality, but not environmental service quality [39]. On the other hand, the direct relationship between satisfaction and trust could also be observed in hotel services [40] and banking services [41]. Various research has revealed the contribution of perceived service quality, satisfaction and trust in forming a patient’s revisit intention [34,37,42]. A study has suggested that perceived service quality has an effect on revisit intention through the mediation of patients’ service value and satisfaction [43], while another study has indicated that satisfaction fosters revisit intention by the psychological attachment of aligning patients’ expectations and the actual services received [44].

This study aimed to explore Hong Kong older adults’ perceptions and experiences of receiving care from their most-visited primary care provider through the PCAT, and to provide directions and insights for primary care providers in Hong Kong to improve service delivery and enhance the utilisation of primary care services by older adults. The proposed research question was: “*How do the experience and perception of primary care affect perceived service quality and satisfaction, and in turn influence the trust and revisit intention among older adults in Hong Kong*?”

## 2. Materials and Methods

### 2.1. Research Hypotheses and Model

#### Research Hypotheses

Patients receive better self-rated care from their GPs in private clinics compared to that from general outpatient clinics, and the difference is mainly attributed to better first contact accessibility and closer patient–doctor relationship with private GPs [45]. A study of the PCAT profile between Hong Kong and Shanghai has found that Hong Kong participants significantly scored higher in first contact utilisation while lower in comprehensiveness of service and ongoing care than Shanghai participants [46]. However, the relationship between perceived service quality and revisit intention among older adults in Hong Kong has not been explored. Despite the importance of strengthening the primary care system, limited research has been conducted to explore the relationship between these constructs in Hong Kong, in particular among older adults, and thus, there remains a research gap. This study aimed to explore the relationship between the aforementioned factors and to answer the research question by the following hypotheses:

**Hypothesis** **1 (H1).** *Perceptions in the seven domains of PCAT (a. first contact accessibility, b. first contact utilisation, c. ongoing care, d. coordination, e. comprehensiveness, f. family-centeredness, g. community orientation) are positively related to the perceived service quality.*

**Hypothesis** **2 (H2).** *Perceptions in the seven domains of PCAT (a. first contact accessibility, b. first contact utilisation, c. ongoing care, d. coordination, e. comprehensiveness, f. family-centeredness, g. community orientation) are positively related to patients’ satisfaction.*

**Hypothesis** **3 (H3).** *Perceived service quality is positively related to patients’ satisfaction.*

**Hypothesis** **4 (H4).** *Perceived service quality is positively related to trust.*

**Hypothesis** **5 (H5).** *Perceived service quality is positively related to revisit intention.*

**Hypothesis** **6 (H6).** *Patients’ satisfaction is positively related to trust.*

**Hypothesis** **7 (H7).** *Patients’ satisfaction is positively related to revisit intention.*

**Hypothesis** **8 (H8).** *Trust is positively related to revisit intention.*

### 2.2. Study Design

**Figure 1:**
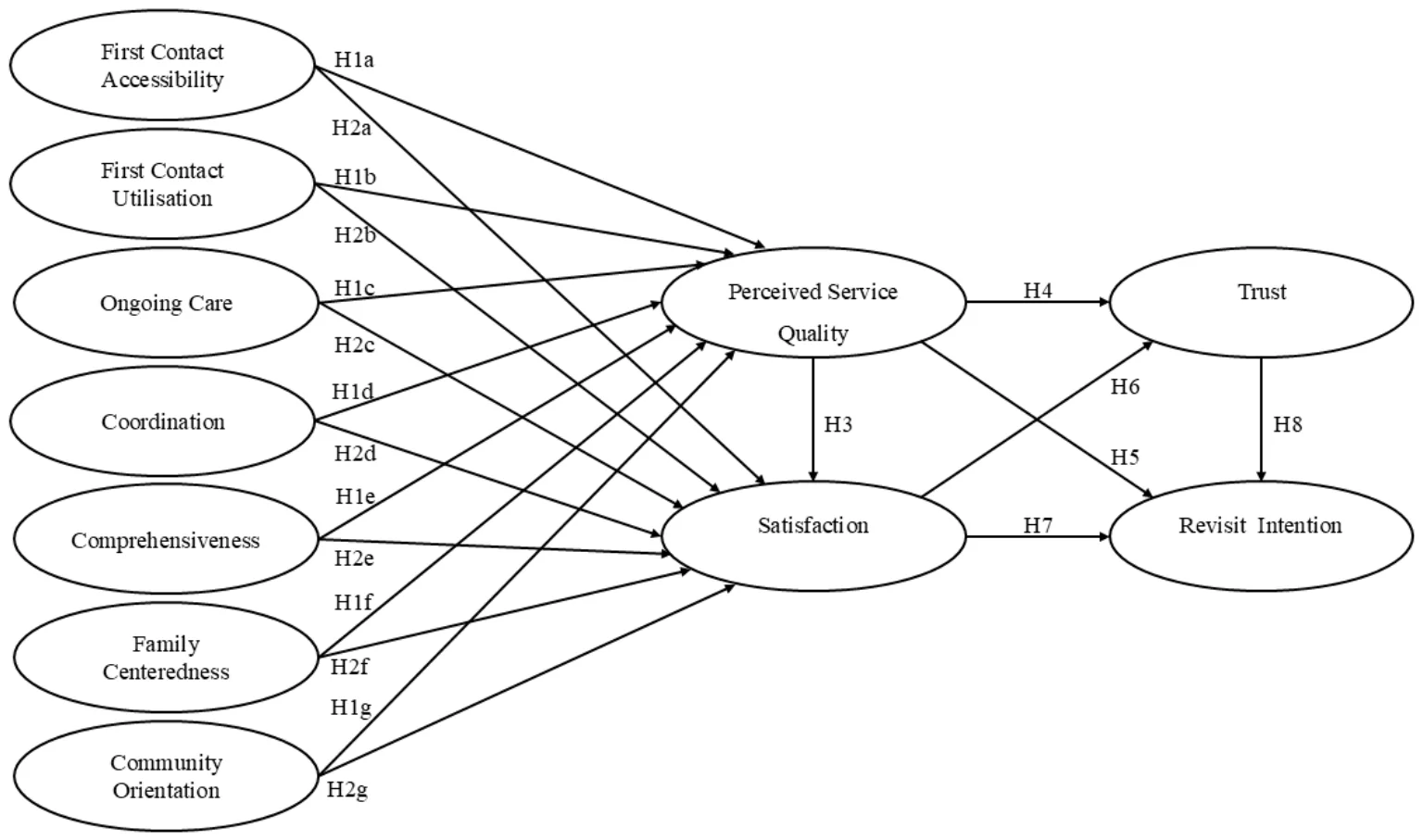
Conceptual model. Source: Authors’ own work.

This was a correlational survey study that adopted cross-sectional design to examine the relationships among the seven PCAT constructs, perceived service quality, satisfaction, trust, and revisit intention as shown in the conceptual framework (Figure 1). In the first stage, the validity and reliability of the PCAT-C were assessed in a pilot study to ensure the compatibility of the PCAT-C with Hong Kong older adults, and details are described in Section 2.4. Based on the results of the pilot study, a questionnaire consisting of 68 items was created for the second stage of this study, and it included two items assessing participants’ eligibility, 51 items measuring participants’ primary care experience, and 15 items collecting socio-demographic and health status information. Participants were asked to complete the questionnaire from their experience with their most frequently visited family doctor. The questionnaire took approximately 15 min to complete. According to the “10-times rule”, the required minimum sample size of a PLS-SEM analysis should be at least ten times the maximum number of structural paths directed at any latent construct in the model [47]. In the conceptual model, the latent variables with the highest number of pointing structural paths are perceived service quality and satisfaction, seven for each variable; therefore, the minimum sample size of this study is 70 (i.e., 7 × 10).

### 2.3. Participants and Sampling

Data collection was performed between March and November 2025. Hong Kong residents aged 60 years or above who reported having a regular source of primary care were eligible for this study. Two screening questions were asked before proceeding to the survey: “Are you a Hong Kong permanent resident who is aged 60 or above?”; “Is there a doctor or clinic that you usually go to when you are sick or need health advice (including Accident and Emergency)?” Participants were recruited using multiple methods, including cluster, purposive, and snowball sampling strategies [48]. Five neighbourhood centres for senior citizens operated by Sik Sik Yuen, a large religious charity organisation, were selected as recruitment sites. These centres represented districts with diverse geographic locations and socio-economic status across Hong Kong of all the 18 districts, and they were located in Shatin, Wong Tai Sin, Yau Tsim Mong, and Kwun Tong. After obtaining permission from the centre directors, centre staff assisted in identifying eligible older adults and inviting them to participate through face-to-face contact, telephone calls, or WhatsApp messages. Interested individuals were then asked to complete the questionnaire at their respective centres with a research assistant (RA) who had been trained for the project. The RA might read out the questions and be available to clarify when needed, but not to influence the answers of the participants. A total of 416 eligible responses were collected from these centres. To recruit more participants, the questionnaire was also circulated through mass email invitations of the university, while distribution to WhatsApp groups was conducted by members of the research team via their personal networks. Recipients were encouraged to share the questionnaire with other older adults within their social networks, thus creating a snowball recruitment process. A total of 155 eligible responses were collected via the snowball recruitment, with respondents coming from all 18 districts of Hong Kong. Additionally, two eligible individuals were purposively approached by the RA in a park in the Yau Tsim Mong district.

Before participation, all participants received information about the purpose and objectives of this study, confidentiality arrangements, and their right to withdraw at any time without consequence. Participants received a gift coupon valued at HK$50 as a token of appreciation. Since the healthcare systems, social services, culture, and demographic characteristics of Hong Kong differ from those of other Chinese regions such as Beijing and Shanghai, such sample selection would limit the generalisability of the results.

### 2.4. Measures

The scale of the PCAT-C adopted in this study was developed and verified from a pilot study. Despite the wide use of the PCAT, limited research using the PCAT to evaluate the experience of primary care has been conducted in Hong Kong, and there was no version of the PCAT tailored for the Hong Kong situation, unlike the K-PCAT in Korea [49] and the ZA PCAT in South Africa [50]. The most related version of the PCAT to Hong Kong’s situation is the PCAT-C, which was first applied on the Chinese population in a study in Changsha, China [51]. However, Hong Kong has certain local elements that might influence the primary care services, including the healthcare system and cultural values. Therefore, to align better with the situation of Hong Kong older adults, a few items in the PCAT-C were modified from the questionnaire adopted in the study by Yang et al. [51]. The face validity of the modified instruments was confirmed by rating the relevance of items by six health experts. Then, the modified version of the PCAT-C was used in the pilot study. The results of the pilot study (n = 26) showed that the internal reliability of the modified PCAT-C was acceptable. There were 7 constructs (Table A1), including first contact utilisation, first contact accessibility, ongoing care, coordination, comprehensiveness, family-centeredness, and community orientation [51].

### 2.5. Data Analysis

IBM SPSS version 30 was used to analyse descriptive statistics, and PLS-SEM using SmartPLS 4.0 was utilised to evaluate the proposed conceptual framework. PLS-SEM is an effective statistical tool in handling a complex structural model, and it can assess the measurement and structural models at once. PLS-SEM’s technical capabilities are highly compatible with this complex conceptual model, allowing the simultaneous examination of all 20 pathways among all 11 latent variables. It is also powerful in predictive and exploratory studies [52] and has been widely used in academic research, with over 10,000 scholars citing the use of SmartPLS [53]. In this study, the reliability (internal reliability) and validity (convergent validity and discriminant validity) of the measurement items were also examined through SmartPLS 4.0. After ensuring reliability and validity, the structural relationships among constructs were analysed through path coefficients.

## 3. Results

### 3.1. Characteristics of Participants

Of the 607 responses collected, 34 were excluded: 29 (4.8%) ineligible participants, two (0.3%) incomplete responses, and three (0.5%) duplicated responses. Consequently, 573 respondents were included in the final analysis. Table 1 presents the participants’ demographic characteristics. A total of 80.5% of the participants were female. The majority of them (52.4%) were aged 70 to 79, followed by those aged 60 to 69 (26.7%). Approximately half (50.7%) had attained a primary level of education or below. Most of them (92.5%) were retired. A total of 59.7% of the participants had less than HK$10,000 in total monthly domestic household income. The distribution of participants from high-income districts (52.5%) and low-income districts (47.5%) was nearly equal. A total of 45.9% of them were married and 35.5% of them were widowed. Over one-third (37.2%) lived alone, while 62.8% of them lived with their spouse and/or their children.

To evaluate the representativeness of the current sample, demographic data on older adults from the 2021 Hong Kong Population Census were extracted and compared to those of the current study [54] (Table A5). However, as the 2021 Population Census defined older adults as individuals aged 65 or above, complete data for the 60–64 age group for certain demographic variables were unavailable. This consequently limited the range of demographics the current study could directly compare with the target population. Among comparable demographic variables (those with consistent or similar categorisation and complete data for the 60–64 age group in the 2021 census report) such as education attainment and household by districts (income), the percentages in the current study and the 2021 census did not differ a lot, suggesting a good representativeness. As for the gender, the percentage of females in the current study was almost 30% higher than that in the 2021 census, while the percentage of widowed in the current study was more than 15% greater than that surveyed in the 2021 census.

### 3.2. Reliability and Validity of the Measurement Model

The reliability and validity of the measurement model were assessed using SmartPLS 4.0. Item removal guidelines based on outer loading were adopted [47]. First, items with outer loadings lower than 0.4 were removed, beginning with the lowest outer loading in each latent variable, until all remaining items had an outer loading of 0.4 or above. Then, items with outer loadings between 0.4 and 0.7 were analysed. These items were removed only if their deletion improved the construct’s average variance extracted (AVE) and composite reliability to meet the recommended threshold. To assess discriminant validity, items with problematic cross-loadings were evaluated and removed when necessary to achieve satisfactory validity. After this refinement process, the remaining items included two items for first contact accessibility, one for first contact utilisation, three for ongoing care, four for coordination, three for comprehensiveness, four for family centeredness, three for community orientation, four for perceived service quality, three for satisfaction, two for trust, and two for revisit intention (Table 2). As first contact utilisation was represented by a single retained item, internal consistency and convergent validity were not assessed for this construct.

The composite reliability for all constructs exceeded the required threshold of 0.7, ranging from 0.807 to 0.944, demonstrating satisfactory internal consistency. For convergent validity, the AVEs of all constructs were greater than 0.5, ranging from 0.512 to 0.893, establishing convergent validity (Table 2). For discriminant validity, both the Fornell–Larcker criterion and the heterotrait–monotrait (HTMT) ratio were assessed (Table 3 and Table 4). For example, the HTMT value between ongoing care and comprehensiveness was 0.436, that between perceived service quality and satisfaction was 0.773, and that between family-centeredness and community orientation was 0.488. The lowest HTMT value was 0.026, noted between first contact utilisation and coordination, reflecting that these two constructs were least related among the relationships among all constructs. The described and all remaining HTMT values were below the recommended threshold of 0.85, except for the relationship between satisfaction and trust (HTMT = 0.87). Given the conceptual similarity of the constructs, the HTMT thresholds of 0.825 and 0.9 were both considered acceptable [55,56]. Satisfaction and trust were treated as separate constructs because they were theoretically distinct and demonstrated adequate discriminant validity according to the Fornell–Larcker criterion (Table 4). For instance, the square root of the AVE of trust (0.945) was higher than the construct correlation between trust and satisfaction (0.724). The square root of the AVE of trust (0.945) was also higher than the construct correlations between trust and first contact accessibility (0.232), first contact utilisation (0.12), ongoing care (0.355), coordination (0.144), comprehensiveness (0.088), family-centeredness (0.141), community orientation (0.235), perceived service quality (0.506), satisfaction (0.724), and revisit intention (0.451). Similarly, all square roots of the AVE for each remaining construct were greater than the correlation coefficients with other constructs, satisfying the Fornell–Larcker criterion. In addition, satisfaction and trust occupied different positions in the structural model, with satisfaction significantly predicting trust (Table 5).

### 3.3. Structural Model

**Figure 2:**
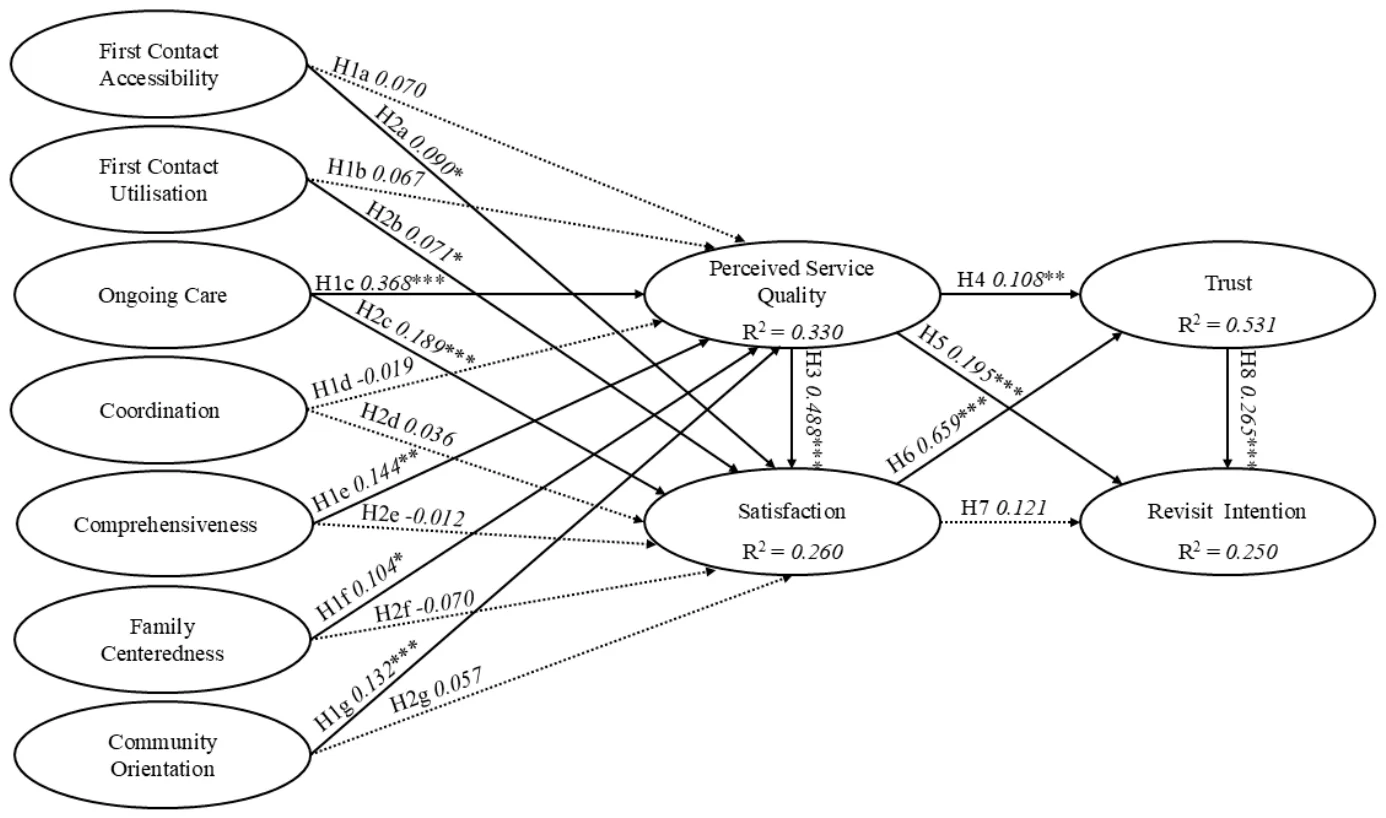
Results of structural modelling (direct effect). Notes: Values besides H1a–H8 are path coefficients for respective hypotheses. R^2^ = R-squared (Coefficient of Determination). * *p* ≤ 0.05, ** *p* ≤ 0.01, *** *p* ≤ 0.001. Significant path—solid arrows. Non-significant path—dashed arrows. Source: Authors’ own work.

The results of the direct effects of the structural model are shown in Table 5. Ongoing care (*β* = 0.368; *p* < 0.0001), comprehensiveness (*β* = 0.144; *p* < 0.01), family-centeredness (*β* = 0.104; *p* < 0.05) and community orientation (*β* = 0.132; *p* < 0.001) were found to have significant positive effects on the perceived service quality. However, there was a non-significant relationship between first contact accessibility and perceived service quality (*β* = 0.070; *p* = 0.052), first contact utilisation and perceived service quality (*β* = 0.067; *p* = 0.091), and coordination and perceived service quality (*β* = −0.019; *p* = 0.676). The results showed that first contact accessibility (β = 0.090; *p* < 0.05), first contact utilisation (*β* = 0.071; *p* < 0.05) and ongoing care (*β* = 0.189; *p* < 0.0001) had significantly positive effect on satisfaction; while coordination (*β* = 0.036; *p* = 0.310, comprehensiveness (*β* = −0.012; *p* = 0.744), family-centeredness (*β* = −0.070; *p* = 0.103), and community orientation (*β* = 0.057; *p* = 0.101) had no significant relationship with satisfaction. Therefore, H1 and H2 were partially supported. In addition, perceived service quality was positively associated with patients’ satisfaction (*β* = 0.488, *p* < 0.0001), trust (*β* = 0.108, *p* < 0.01), and revisit intention (*β* = 0.195, *p* = 0.001). It was also found that trust (*β* = 0.265; *p* < 0.0001) was positively correlated with revisit intention. However, patients’ satisfaction was positively related to trust (*β* = 0.659; *p* < 0.0001) but not revisit intention (*β* = 0.121, *p* = 0.082). The results of the structural model are shown in Figure 2. Age and gender were added in the structural model as control variables to test their potential effects on revisit intention, and no significant impact was found (age with *β* = 0.003, *ns*; gender with *β* = −0.021, *ns*). Overall, the added control variables did not alter the significance of the hypothesised relationships and thus provided additional support for the stability and robustness of the results.

Results of the mediation model are shown in Table 6. Trust had a partial mediating effect between perceived service quality and revisit intention (*β* = 0.028, *p* = 0.032), showing that perceived service quality influenced revisit intention both directly and indirectly through trust. Trust exerted an indirect-only effect on the relationship between satisfaction and revisit intention (*β* = 0.174, *p* < 0.001). It meant that satisfaction influenced revisit intention primarily through trust. Satisfaction had a partial mediating effect on the association between perceived service quality and trust (*β* = 0.325, *p* < 0.001). A significant sequential mediation effect had thus been found, with perceived service quality influencing revisit intention indirectly through satisfaction and trust in sequence (*β* = 0.086, *p* < 0.001). These findings demonstrated the central role of trust as a key construct linking perceived service quality and satisfaction to revisit intention. As sociodemographic factors might influence the pathways, multi-group analysis (MGA) comparisons were performed, and the pathways significantly influenced by specific factors are listed in Table A3 of Appendix A.

## 4. Discussion

This study has shown that the perceived service quality is significantly influenced by ongoing care, comprehensiveness, community orientation, and family-centeredness. Consistent with the previous literature [57,58], the findings confirm that ongoing care is the most salient predictor of perceived service quality. As older adults commonly navigate complex multimorbidity, ongoing care minimises repetitive history-taking and fosters greater security and trust [57]. The positive relationship between perceived service quality and satisfaction in this study is consistent with previous studies [29,59,60]. Older adults frequently utilise primary care for the continuing management of chronic conditions and age-related vulnerabilities, and their continuous interaction with the healthcare system makes them highly sensitive to the quality of service provided. Addressing psychosocial and functional needs in primary care enhances older adults’ sense of respect, safety, and recognition, yielding higher perceived quality and increased patient satisfaction [61,62].

Moreover, perceived service quality and trust have been found to be significantly associated with revisit intention in this study, and this is in line with previous studies [34,63]. Older adults who trust their primary care providers are more likely to return for regular follow-ups and consult their primary care physician first when a new health issue arises, thereby enabling timely disease detection, ongoing care, and more effective management of chronic conditions as well as acute ailments. Studies have suggested that patients’ trust in doctors is built from their perception of the healthcare quality provided by the doctor, and a key to enhancing perceived healthcare quality is to establish effective communication. By clearly explaining the treatment and illnesses, patients are more likely to perceive their doctor as having high healthcare quality and therefore building the trust [36]. Moreover, the vital mediating role of trust in linking perceived service quality and revisit intention has also been supported by several healthcare studies [28,64].

Intentions to return to a primary care provider by older adults is rarely determined by clinical metrics alone. It hinges on the quality of the therapeutic relationship, and the security fostered throughout the care process, such as empathy of the medical staff, clarity of communication, and reliability of administrative processes [57]. Consistent with recent evidence [65], perceived service quality in healthcare extends beyond clinical effectiveness because perceived empathy and trustworthiness directly and indirectly influence patient revisit intention.

The findings of this study suggest that family-centeredness is positively related to perceived service quality, which in turn positively influences revisit intention. This mechanism is consistent with previous research on inpatient behaviours, which indicates that a patient’s revisit intention is heavily influenced by their family members’ perceptions of the healthcare provider. To be precise, when medical staff are perceived by family members as possessing high perceived service quality (e.g., demonstrating high clinical competence), patients exhibit a greater willingness to revisit the doctor [65].

Contrary to expectations, first contact accessibility, the ease of access of a physical visit, is not found to have a significant direct influence on perceived service quality. The lack of a significant relationship between first contact accessibility and perceived service quality is attributed to the geographical uniqueness of Hong Kong, characterised by its limited spatial extent and a high density of approximately 5000 private clinics. A recent study has found that 95% of Hong Kong citizens can reach primary healthcare from their home within 15 min, a uniquely short travel time globally [66]. Consequently, this dense distribution creates a ceiling effect, significantly diminishing the impact of spatial accessibility on the overall perceived service quality.

In addition, the present study has found that family-centeredness has not significantly affected older adults’ overall satisfaction or their perceived service quality in primary care. Generational differences heavily influence patient expectations. Compared to younger populations, older adults consistently report higher baseline satisfaction with healthcare encounters. They also prefer less control or involvement in medical decisions, and they tend to let a familiar healthcare provider make most of the decisions [67], suggesting that neither their own involvement nor their family members’ participation is highly valued. While family involvement is often assumed to be universally beneficial, recent qualitative literature highlights that older adults frequently report feeling that family caregivers exaggerate their medical conditions, leading to contentious visit dynamics [68].

The absence of a positive association between coordination and satisfaction may reflect entrenched care-seeking habits among local residents. In Hong Kong, patients commonly prefer direct access to specialists without first consulting a family doctor, limiting their exposure to coordinated primary care [69]. In particular, older adults use more private primary care for acute episodes because they can use the Elderly Health Voucher Scheme [70] when they feel sick, including illnesses they thought could only be cured by specialists. This subsidy scheme enlarges their autonomy of making a direct visit to a specialist. Participants might not expect the coordination of services to be part of family doctors’ responsibilities. Therefore, the elements of coordination, including discussing the treatment plan with family doctors, writing a referral letter, and follow-up of the specialist referral by family doctors may not be applicable to Hong Kong citizens.

### 4.1. Practical Implications

This study has addressed a research gap by examining how different dimensions of primary care, as measured by the PCAT, are associated with perceived service quality and satisfaction among older adults in Hong Kong. The findings provide actionable insights for the organisation, management and delivery of primary care services, particularly for the older adult population. To improve perceived service quality and satisfaction, healthcare providers should consider prioritising ongoing care, given the high prevalence of multimorbidity among older adults. District Health Centres are uniquely positioned to lead these efforts by enabling older adults to develop sustained and trust-based relationships with familiar primary care providers.

Furthermore, addressing the critical need for comprehensiveness requires that District Health Centres continue to strengthen their cross-disciplinary networks. By integrating medical consultations, allied health services, and psychosocial support within a coordinated model, District Health Centres can function as community-based health hubs. To foster revisit intentions, building trust and enhancing the perceived service quality of primary care is important and healthcare professionals are recommended to confirm that clinical expertise is paired with strong interpersonal and communication skills, including empathy and patient-centred engagement. Aligning with prior research [71], strengthening these soft skills has the potential to substantially improve older adults’ healthcare experiences and perceived quality of care.

The ease of first contact accessibility has always been seen as indispensable in high-performing primary care [9]. A recent study has even suggested people in semi-rural or rural areas value first contact accessibility more than the quality of care received because of limited hospital choice [72]. However, a complete understanding of it from the patient’s perspective may still be lacking. In light of the lack of a significant relationship between first contact accessibility and perceived service quality, this study has suggested that, in big cities like Hong Kong, characterised by a mature transportation system, the ease of access is often less of an issue, but people’s willingness to revisit a family doctor depends on other factors like their level of trust and perceived service quality.

Additionally, several concrete, actionable strategies are advised for the various stakeholders in the primary care sector. For primary care managers, it is suggested to integrate perceived service quality and trust into frontline staff training protocols. Such training may consider explicitly instructing practitioners to provide comprehensive, easily understood explanations regarding a patient’s illness and treatment plan, aiming to demonstrate professional clinical conduct and establish a foundation of trust. To further enhance the ongoing quality of care within private clinic settings, healthcare providers could consider implementing proactive follow-up protocols. For instance, clinic nurses or family doctors could contact patients near their anticipated recovery date, estimated by the duration of the prescribed medication course, as a follow-up consultation. To enhance informational continuity, family doctors could review patients’ medical records prior to the consultation and actively start the consultation with specific inquiries about the patient’s progress since their last visit, regardless of whether the patient raises concerns or relevant issues. Moreover, for private clinics operating with multiple GPs on shift, instead of randomly assigning patients to available time slots, administrative staff could actively offer patients the opportunity to consult with their preferred GP, or the one they visit most frequently, which fosters relational continuity.

### 4.2. Limitations

One limitation of this study relates to participant eligibility. As only older adults in Hong Kong with a regular primary care provider were included, the findings in this paper may not be generalised to those without a regular primary care provider, including those who engage in “doctor-shopping” or who seldom utilise primary care services. Additionally, a proportion of the data collection was conducted at local neighbourhood centres, where participants were members. As a result, participants from neighbourhood centres may possess greater health knowledge than the general population, as these centres often hold health talks. Bias may occur because they may have a more complete understanding of the family doctor’s role and apply stricter criteria when rating their satisfaction. Also, participants from neighbourhood centres are likely to have a higher degree of social connection and community engagement than the general older population. With a weaker social safety net, they may be more likely to seek their family doctors as a primary source of support during illness. This may foster greater social and emotional dependence on their doctors. Therefore, their baseline perspective on family doctors may differ from that of socially engaged older adults, potentially manifesting as more unconditional trust. Caution should be exercised when generalising the results to the broader older population, particular to older adults with limited social engagement.

Furthermore, apart from a potential higher social engagement, the religiously oriented character of Sik Sik Yuen, which equally values Taoism, Buddhism, and Confucianism, may have also exposed participants recruited from its neighbourhood centre to a greater influence of religion than the general older population. Influenced by religious values that teach interpersonal trust as an essential virtue to be pursued, these participants may exhibit a higher baseline of trust toward others, including their GPs, thereby fostering a stronger predisposition toward revisit intentions. Therefore, findings should be generalised cautiously to the older population with no religious belief or those with diversified backgrounds of religious belief.

When inviting participants from different districts, caution was exercised to minimise potential sampling bias. Specifically, the research team sought to avoid an overrepresentation of participants from districts with a particular socioeconomic level, which could create an imbalanced sample skewed by household income. A notable potential bias is the confounding effect this could have on satisfaction. As a bibliometric analysis has pointed out, the cost of medical care substantially affects patient satisfaction [16]. Since individuals residing in high-socioeconomic districts are generally wealthier than those in low-socioeconomic districts, a sample skewed toward the latter may exhibit a lower baseline satisfaction with GP consultations, as their cost is relatively higher, and vice versa. Regardless of the direction of the skew, this imbalance introduces a confounding variable that could distort the results of hypothesis testing involving patient satisfaction. The sample was also predominantly female (80.5%), and no data were collected from older adults residing in residential care homes or nursing homes. The discrepancies in the gender and marriage status between the sample and the census population also limit the generalisability of the results, and caution should be taken when generalising the results to the older adult population in Hong Kong. Further studies should examine experiences and perceptions of the underrepresented population (the males, people who lack a usual source of care, people living in an assisted living facility, and in particular, people who have little health knowledge and people who lack an active social network). Furthermore, as part of the data was collected online, potential self-selection bias may limit this study’s generalisability. The actual differences between the face-to-face survey and online survey on each pathway were tested through MGA comparison and shown in Table A6 in Appendix A. In fact, significant difference only occurs in the pathway from satisfaction to trust.

Furthermore, as the data of exogenous variables and endogenous variables were collected at one time in the same questionnaire, potential single method bias may inflate or deflate the validity and reliability of the item in use. In addition, because outer loading refinement pruned a PCAT construct (first contact utilisation) to single-item measure, results should be interpreted with caution, as a single-item measure may not fully capture its correlation with other constructs. Moreover, further refinement of the PCAT should also be considered for future studies, including modifying items with ambiguous and complex wordings, and removing items with obvious responses or implicit assumptions that may not be compatible with Hong Kong’s situation. In addition, future work could consider exploring in detail how the identified factors influence the perceived service quality, satisfaction, trust, and revisit intention among Hong Kong older adults by performing qualitative studies such as focus group discussion and case study. A triangulation analysis of such qualitative data with the current study’s quantitative data may further bring a comprehensive view on older adults’ perception of primary care. Moreover, the present findings provide deeper insights into the interrelated relationships among perceived service quality, satisfaction, trust, and revisit intention as distinct concepts. This study extends the literature on behavioural intentions by incorporating psychological determinants that are scarcely studied in traditional healthcare utilisation frameworks, such as the Andersen’s Behavioural Model of Health Services Use. Future research can build upon these insights to further examine these dynamics across diverse healthcare settings and complement existing models in explaining healthcare utilisation.

## 5. Conclusions

In summary, this study has provided a comprehensive understanding of older adults’ perception of key domains of primary care in Hong Kong and how their experience may influence their intention to revisit the same family doctor. The findings have highlighted the central role of perceived service quality and trust in shaping the utilisation of primary care by older adults of Hong Kong. In congruence with prior research, strengthening trust in primary care providers and enhancing perceived service quality are therefore critical priorities. Aligning with prior research suggesting the contribution of ongoing care to trust and perceived service quality, as well as customer loyalty to revisit intentions, one promising approach is to reinforce ongoing care within primary care practices. By promoting ongoing and sustained patient–provider relationships, revisit intentions may be increased, leading to more stable utilisation of primary care services among older adults in Hong Kong.

## Figures and Tables

**Table 1:** Demographic characteristics of participants.

	Frequency (*N* = 573)	(%)
*Gender*		
Male	112	19.5
Female	461	80.5
*Age*		
60–69	153	26.7
70–79	300	52.4
80–89	102	17.8
90 or above	18	3.1
*Education Attainment*		
Primary or below	291	50.7
Secondary	189	33.0
Diploma	36	6.3
Bachelor’s degree	29	5.1
Master’s degree or above	28	4.9
*Employment Status*		
Full time	17	3.0
Part time	24	4.2
Unemployed	2	0.3
Retired	530	92.5
*Total Monthly Domestic Household Income*		
Less than HK$10,000	342	59.7
HK$10,000–HK$20,000	105	18.3
HK$20,001–HK$30,000	58	10.1
More than HK$30,000	68	11.9
*Household by Districts (income)*		
High-income district	301	52.5
Low-income district	272	47.5
*Marriage Status*		
Single	61	10.6
Married	263	45.9
Divorced	46	8.0
Widowed	203	35.5
*Living Arrangement*		
Living alone	213	37.2
Living with spouse	169	29.5
Living with spouse and child(ren)	85	14.8
Living with child(ren) only	106	18.5

Source: Authors’ own work.

**Table 2:** Reliability and validity of the measurement model—retained items.

Constructs and Items	Mean	SD	Loadings	AVE	Composite Reliability
*First Contact Accessibility*				0.635	0.775
First Contact Accessibility_ item1	3.620	1.433	0.870		
First Contact Accessibility_item2	2.175	1.307	0.716		
*Ongoing Care*				0.637	0.840
Ongoing care_item1	4.007	1.062	0.788		
Ongoing care_item2	3.281	1.383	0.786		
Ongoing care_item3	3.368	1.471	0.821		
*Coordination*				0.512	0.807
Coordination_item1	2.590	1.612	0.757		
Coordination_item2	3.627	1.496	0.726		
Coordination_item3	2.656	1.462	0.741		
Coordination_item4	2.782	1.536	0.630		
*Comprehensiveness*				0.682	0.865
Comprehensiveness_item1	3.150	1.675	0.875		
Comprehensiveness_item2	2.269	1.567	0.805		
Comprehensiveness_item3	3.255	1.664	0.796		
*Family-centeredness*				0.550	0.830
Family-centeredness_item1	1.743	1.184	0.743		
Family-centeredness_item2	1.855	1.352	0.793		
Family-centeredness_item3	2.934	1.655	0.733		
Family-centeredness_item4	2.194	1.431	0.695		
*Community orientation*				0.702	0.876
Community orientation_item1	2.487	1.241	0.831		
Community orientation_item2	2.227	1.157	0.838		
Community orientation_item3	2.180	1.065	0.844		
*Perceived service quality*				0.543	0.826
Perceived service quality_item1	3.063	1.272	0.721		
Perceived service quality_item2	2.897	1.310	0.733		
Perceived service quality_item3	3.688	0.857	0.746		
Perceived service quality_item4	3.818	0.746	0.749		
*Satisfaction*				0.700	0.875
Satisfaction_item1	3.864	0.725	0.870		
Satisfaction_item2	3.731	0.700	0.834		
Satisfaction_item3	3.757	0.683	0.805		
*Trust*				0.893	0.944
Trust_item1	3.967	0.594	0.945		
Trust_item2	3.948	0.583	0.945		
*Revisit Intention*				0.710	0.830
Revisit Intention_item1	3.979	0.716	0.875		
Revisit Intention_item2	3.770	0.919	0.808		

Notes: SD, standard deviation; Loadings, outer loadings; AVE, average variance extracted. Source: Authors’ own work.

**Table 3:** Discriminant validity (heterotrait–monotrait method).

	Ac	Ut	OC	Coo	Com	FC	CO	PSQ	Sa	Tt	RI
First Contact Accessibility (Ac)	–										
First Contact Utilisation (Ut)	0.116	–									
Ongoing Care (OC)	0.278	0.17	–								
Coordination (Coo)	0.141	0.026	0.369	–							
Comprehensiveness (Com)	0.062	0.053	0.436	0.54	–						
Family-Centeredness (FC)	0.085	0.035	0.393	0.756	0.765	–					
Community Orientation (CO)	0.244	0.071	0.359	0.24	0.347	0.488	–				
Perceived Service Quality (PSQ)	0.282	0.153	0.677	0.323	0.513	0.48	0.42	–			
Satisfaction (Sa)	0.364	0.199	0.613	0.241	0.268	0.249	0.321	0.773	–		
Trust (Tt)	0.375	0.128	0.446	0.191	0.101	0.177	0.27	0.609	0.87	–	
Revisit intention (RI)	0.144	0.235	0.377	0.257	0.181	0.195	0.182	0.605	0.621	0.613	–

Notes: Values included are heterotrait–monotrait ratio of correlations. Source: Authors’ own work.

**Table 4:** Discriminant validity (Fornell–Larcker criterion method).

	Ac	Ut	OC	Coo	Com	FC	CO	PSQ	Sa	Tt	RI
First Contact Accessibility (Ac)	***0.797***										
First Contact Utilisation (Ut)	*0.080*	***1***									
Ongoing Care (OC)	*0.175*	*0.142*	***0.798***								
Coordination (Coo)	*0.058*	*0.023*	*0.276*	***0.715***							
Comprehensiveness (Com)	*0.002*	*−0.003*	*0.328*	*0.42*	***0.826***						
Family-Centeredness (FC)	*0.041*	*0.031*	*0.283*	*0.551*	*0.575*	***0.741***					
Community Orientation (CO)	*0.149*	*0.067*	*0.288*	*0.184*	*0.276*	*0.37*	***0.838***				
Perceived Service Quality (PSQ)	*0.158*	*0.136*	*0.499*	*0.23*	*0.353*	*0.335*	*0.327*	***0.736***			
Satisfaction (Sa)	*0.215*	*0.174*	*0.461*	*0.174*	*0.213*	*0.186*	*0.267*	*0.605*	***0.836***		
Trust (Tt)	*0.232*	*0.12*	*0.355*	*0.144*	*0.088*	*0.141*	*0.235*	*0.506*	*0.724*	***0.945***	
Revisit intention (RI)	*0.080*	*0.177*	*0.246*	*0.15*	*0.08*	*0.116*	*0.131*	*0.402*	*0.431*	*0.451*	***0.842***

Notes: Construct correlations (in italic). Square roots of average variance extracted (bold and italic). Source: Authors’ own work.

**Table 5:** Results of structural modelling (direct effect).

	Path Relations	*β*	*t* (*p*)	*f* ^2^	95% CI	Outcome
H1a	First Contact Accessibility → Perceived Service Quality	0.070	1.947 (*p* = 0.052)	0.006	[−0.004, 0.135]	Rejected
H1b	First Contact Utilisation → Perceived Service Quality	0.067	1.689 (*p* = 0.091)	0.006	[−0.011, 0.146]	Rejected
H1c	Ongoing Care → Perceived Service Quality	0.368	9.509 (*p* = 0.000)	0.157	[0.291, 0.441]	Supported
H1d	Coordination → Perceived Service Quality	−0.019	0.418 (*p* = 0.676)	0	[−0.108, 0.07]	Rejected
H1e	Comprehensiveness → Perceived Service Quality	0.144	3.030 (*p* = 0.002)	0.022	[0.049, 0.237]	Supported
H1f	Family-Centeredness → Perceived Service Quality	0.104	2.078 (*p* = 0.038)	0.009	[0.005, 0.204]	Supported
H1g	Community Orientation → Perceived Service Quality	0.132	3.295 (*p* = 0.001)	0.021	[0.055, 0.214]	Supported
H2a	First Contact Accessibility → Satisfaction	0.090	2.453 (*p* = 0.014)	0.019	[0.017, 0.162]	Supported
H2b	First Contact Utilisation → Satisfaction	0.071	2.021 (*p* = 0.043)	0.014	[0.003, 0.142]	Supported
H2c	Ongoing Care → Satisfaction	0.189	4.508 (*p* = 0.000)	0.147	[0.1, 0.266]	Supported
H2d	Coordination → Satisfaction	0.036	1.015 (*p* = 0.310)	0.001	[−0.038, 0.105]	Rejected
H2e	Comprehensiveness → Satisfaction	−0.012	0.327 (*p* = 0.744)	0.003	[−0.085, 0.058]	Rejected
H2f	Family-Centeredness → Satisfaction	−0.070	1.630 (*p* = 0.103)	0	[−0.155, 0.014]	Rejected
H2g	Community Orientation → Satisfaction	0.057	1.640 (*p* = 0.101)	0.016	[−0.014, 0.123]	Rejected
H3	Perceived Service Quality → Satisfaction	0.488	11.241 (*p* = 0.000)	0.278	[0.399, 0.57]	Supported
H4	Perceived Service Quality → Trust	0.108	2.628 (*p* = 0.009)	0.015	[0.027, 0.188]	Supported
H5	Perceived Service Quality → Revisit Intention	0.195	3.292 (*p* = 0.001)	0.031	[0.075, 0.308]	Supported
H6	Satisfaction → Trust	0.659	15.771 (*p* = 0.000)	0.595	[0.575, 0.738]	Supported
H7	Satisfaction → Revisit Intention	0.121	1.740 (*p* = 0.082)	0.008	[−0.007, 0.262]	Rejected
H8	Trust → Revisit Intention	0.265	3.940 (*p* = 0.000)	0.044	[0.125, 0.391]	Supported

Notes: *B*, Beta Coefficient; *t*, t-values; *p*, *p*-values; *f*^2^, f-squared; 95% CI, bias-corrected 95% confidence intervals. Source: Authors’ own work.

**Table 6:** Results of mediation analysis.

Path Relations	Indirect Effect	*t*	*p*	Result
Perceived Service Quality → Trust → Revisit Intention	0.028	2.140	0.032	Partial mediating effect
Satisfaction → Trust → Revisit Intention	0.174	3.816	0.000	Indirect-only effect
Perceived Service Quality → Satisfaction → Trust	0.325	9.223	0.000	Partial mediating effect
Perceived Service Quality → Satisfaction → Trust → Revisit Intention	0.086	3.514	0.000	Partial mediating effect

Notes: *t*, t-values; *p*, *p*-values. Source: Authors’ own work.

## Data Availability

The data presented in this study are available on request from the corresponding author. The data are not publicly available due to ethical considerations; the dataset contains demographic information and potential personal identifiers of the participants.

## Appendix A

**Table A1:** Definition of the primary care constructs of PCAT-C.

Primary Care Construct	Definition	Definition Adopted From
First contact utilisation	Patients’ initial decision to seek care from a primary healthcare provider when a health problem occurs	[73]
First contact accessibility	Provision of equitable and timely access to healthcare services	[9]
Ongoing care	The continuous services offered by primary care providers and includes both relational continuity (whether patients are consistently seen by the same care provider) and informational continuity (whether the provider has access to the patient’s most updated medical records)	[74]
Coordination	Appropriate referral from a primary care provider to other healthcare professionals, such as specialists, to cope with patient’s specific health needs	[74]
Comprehensiveness	The breadth of healthcare knowledge, services, and advice given by the healthcare provider in managing a wide range of patient conditions	[74]
Family-centeredness	Emphasises the participation of family members in health assessment and the consideration of patient’s family medical history	[73]
Community orientation	The acknowledgement of community healthcare needs through direct visit, assessing services provided, and conducting healthcare survey in the community	[75]

Source: Authors’ own work.

**Table A2:** Retained items of the PCAT, perceived service quality, satisfaction, trust, and revisit intention construct.

**First Contact Utilisation**
4. When you have a new health problem, do you go to your GP/family doctor before going somewhere else? (Ut1)
**First Contact Accessibility**
6. Do you inquire over the phone when your GP/family doctor’s clinic is open? (Ac1)
7. Do you inquire over the phone when your GP/family doctor’s clinic is closed? (Ac2)
**Ongoing Care**
10. Are your questions answered in ways that you understand? (OC1)
11. Do you talk to the doctor who knows you best if you have questions? (OC2)
12. Does your GP/family doctor give you enough time and make you feel comfortable talking about your concerns? (OC3)
**Coordination**
17. Does your GP/family doctor discuss with you about different places, recommend a better doctor/place and illustrate the reasons to get help for your problem? (Coo1)
18. Does your GP/family doctor write down any information for the specialist about the reason for the visit? (Coo2)
19. Does your GP/family doctor talk with you about what has happened at the visit and know the result of your visit to the specialist? (Coo3)
20. Does your GP/family doctor help you to make appointment to see a specialist? (Coo4)
**Comprehensiveness**
21. Does your GP/family doctor give you advice about healthy lifestyle, e.g., diet and enough sleep? (Com1)
22. Does your GP/family doctor give you advice about home safety, such as air circulation or passage space? (Com2)
25. Does your GP/family doctor give you advice about exercise? (Com3)
**Family-Centeredness**
32. Does your GP/family doctor ask about your ideas about planning treatment for you or your family members? (FC1)
33. Does your GP/family doctor introduce to you and your family the types of medicines you could possibly get and ask about your ideas before giving a prescription? (FC2)
34. Does your GP/family doctor ask about illness or problems that might run in your family? (FC3)
35. Does your GP/family doctor meet with members of your family if needed? (FC4)
**Community Orientation**
37. Does your GP/family doctor know about important health problems of your community? (CO1)
38. Does your GP/family doctor make a survey of patients to see whether needs were met? (CO2)
39. Does your GP/family doctor conduct a survey of your community to find out health problems? (CO3)
**Perceived Service Quality**
40. The GP/family doctor I usually go to gives me detailed information about my illnesses. (PSQ1)
41. The GP/family doctor I usually go to gives me complete information about my illnesses. (PSQ2)
42. The GP/family doctor I usually attend always solves my health problems. (PSQ3)
43. The GP/family doctor that I usually attend gives me security regarding my health. (PSQ4)
**Satisfaction**
45. Overall, I am very satisfied with my experience at this GP/family doctor. (Sa1)
46. My decision to visit this GP/family doctor for healthcare service was a wise one. (Sa2)
47. Overall, compared to other GPs/family doctors in various places, I am satisfied with this GP/family doctor. (Sa3)
**Trust**
49. I feel I can trust this GP/family doctor. (Tt1)
50. I think that medical quality and services at this GP/family doctor are of high integrity. (Tt2)
**Revisit Intention**
51. I want to continue consulting the same GP/family doctor from now on. (RI1)
52. I would consult my GP/family doctor first when I develop a new health problem. (RI2)

Notes: GP = general practitioner. Source: Authors’ own work.

**Table A3:** Results of bootstrap MGA on demographic variables.

	Groups	Specific Pathways	*p*-Value
Gender	/	/	
Age	70–79 vs. 80 or above	Com → Sa	0.015
		Sa → Tt	0.028
Education Attainment	Secondary or below vs. diploma or above	CO → PSQ	0.006
		FC → PSQ	0.003
Employment status	/		
Total monthly domestic household income	Less than HK$10,000 vs. HK$10,001–20,000	Com → Sa	0.039
		PSQ → RI	0.035
	Less than HK$10,000 vs. HK$20,001–30,000	CO → PSQ	0.033
	Less than HK$10,000 vs. More than HK$30,000	Ac → Sa	0.017
		CO → Sa	0.028
Household by districts (income)	Low-income district vs. High-income district	Ut → PSQ	0.025
Marital status	Divorced vs. Married	Coo → Sa	0.013
		OC → Sa	0.016
	Divorced vs. Single	Coo → Sa	0.024
		PSQ → Tt	0.012
		Sa → Tt	0.015
	Divorced vs. Widowed	Coo → Sa	0.025
		Sa → Tt	0.022
	Married vs. Single	OC → Sa	0.008
		PSQ → Tt	0.014
		Sa → Tt	0.020
	Married vs. Widowed	Sa → Tt	0.015
	Single vs. Widowed	FC → PSQ	0.029
Living arrangement	Living Alone vs. Living with children only	CO → PSQ	0.028
		FC → Sa	0.008
		Ut → Sa	0.002
	Living Alone vs. Living with spouse and children	Com → Sa	0.029
		Sa → RI	0.025
		Sa → Tt	0.008
		Tt → RI	0.004
	Living with children only vs. Living with spouse	CO → PSQ	0.013
		Ut → Sa	0.007
	Living with children only vs. Living with spouse and children	Com → PSQ	0.042
		OC → Sa	0.030
		Sa → Tt	0.032
	Living with spouse vs. Living with spouse and children	Sa → Tt	0.029
		Tt → RI	0.048
		Ut → PSQ	0.041

Notes: Ac = first contact accessibility; Ut = first contact utilisation; OC = ongoing care; Coo = coordination; Com = comprehensiveness; FC = family-centeredness; CO = community orientation; PSQ = perceived service quality; Sa = satisfaction; Tt = trust; RI = revisit intention. Source: Authors’ own work.

**Table A4:** Results of MGA comparison (gender).

	Difference (Female—Male)	1-Tailed (Female vs. Male) *p* Value	2-Tailed (Female vs. Male) *p* Value
First Contact Accessibility → Perceived Service Quality	0.057	0.302	0.604
First Contact Accessibility → Satisfaction	0.033	0.352	0.705
Community Orientation → Perceived Service Quality	0.027	0.408	0.817
Community Orientation → Satisfaction	−0.009	0.531	0.938
Comprehensiveness → Perceived Service Quality	0.067	0.299	0.599
Comprehensiveness → Satisfaction	0.183	0.041	0.081
Coordination → Perceived Service Quality	0.178	0.078	0.157
Coordination → Satisfaction	−0.056	0.671	0.657
Family Centeredness → Perceived Service Quality	−0.202	0.914	0.171
Family Centeredness → Satisfaction	0.002	0.496	0.991
Ongoing Care → Perceived Service Quality	−0.083	0.825	0.350
Ongoing Care → Satisfaction	−0.087	0.760	0.480
Perceived Service Quality → Revisit Intention	0.021	0.443	0.885
Perceived Service Quality → Satisfaction	−0.033	0.635	0.730
Perceived Service Quality → Trust	0.051	0.288	0.575
Satisfaction → Revisit Intention	−0.432	0.933	0.133
Satisfaction → Trust	−0.126	0.918	0.165
Trust → Revisit Intention	0.461	0.036	0.072
First Contact Utilisation → Perceived Service Quality	0.023	0.401	0.802
First Contact Utilisation → Satisfaction	−0.072	0.772	0.456

Source: Authors’ own work.

**Table A5:** Demographic characteristics of participants in 2021 Population Census.

	Frequency (*N* = 2,065,316/1,020,356/2,065,146/1,451,514)	(%)
*Gender* ^		
Male	982,560	47.6
Female	1,082,756	52.4
*Age* ^		
60–69	1,101,218	53.5
70–79	567,867	27.5
80–84	165,172	8
85 or above	226,210	11
*Education Attainment* ^		
Primary or below	953,553	46.2
Secondary	853,816	41.3
Diploma	113,856	5.5
Bachelor’s degree	107,024	5.2
Master’s degree or above	37,067	1.8
*Employment Status* ^		
Employed	478,677	23.2
Economically Inactive Population	120,766	5.8
Retired	1,267,417	61.4
Others	198,456	9.6
*Total Monthly Domestic Household Income* ^^		
Less than HK$10,000	523,755	51.4
HK$10,000–HK$19,999	141,728	13.9
HK$20,000–HK$29,999	222,747	21.8
HK$30,000 or above	132,126	12.9
*Household by Districts (income)* ^		
High-income district	1,072,599	51.9
Low-income district	992,547	48.1
*Marriage Status* ^		
Single	137,613	6.7
Married	1,421,563	68.9
Divorced	133,368	6.4
Widowed	372,772	18
*Living Arrangement* ^^		
Living alone	188,569	13
Living with spouse	396,068	27.3
Living with spouse and child(ren)	458,338	31.6
Living with child(ren) only	265,640	18.3
Others	73,861	5.1
Living in non-domestic household	69,038	4.8

Source: 2021 Population Census [54] and authors’ work (recombination of graphs and tables). Aside from *Gender*, *Age*, and *Education Status*, the categorization of the remaining demographic variables in Table A5 differs from that in Table 1. This discrepancy arises because the categories used in the 2021 Population Census data do not align with or directly correspond to the levels of categorization used in the current study. For *Gender, Age, Education Status, Employment Status, and Marriage Status*, *N* = 2,065,316; for *Total Monthly Domestic Household Income*, *N* = 1,020,356; for *Household by Districts (income)*, *N* = 2,065,146; for *Living Arrangement*, *N* = 1,451,514. ^ Population aged 60 or above; ^^ population aged 65 or above. High-income districts: Central and Western, Tsuen Wan, Wan Chai, Sai Kung, Southern, Eastern, Sha Tin, Yau Tsim Mong, Tai Po, Kowloon City, and Islands; low-income districts: Kwai Tsing, Sham Shui Po, Wong Tai Sin, Kwun Tong, Tuen Mun, Yuen Long, and North.

**Table A6:** Results of MGA comparison (face-to-face survey vs. online survey).

	Difference (Online Survey—Face-to-Face Survey)	1-Tailed (Online Survey vs. Face-to-Face Survey) *p* Value	2-Tailed (Online Survey vs. Face-to-Face Survey) *p* Value
First Contact Accessibility → Perceived Service Quality	0.012	0.437	0.875
First Contact Accessibility → Satisfaction	0.040	0.311	0.623
Community Orientation → Perceived Service Quality	−0.090	0.851	0.297
Community Orientation → Satisfaction	0.069	0.210	0.420
Comprehensiveness → Perceived Service Quality	−0.193	0.966	0.068
Comprehensiveness → Satisfaction	0.005	0.475	0.950
Coordination → Perceived Service Quality	0.036	0.347	0.693
Coordination → Satisfaction	−0.010	0.547	0.906
Family Centeredness → Perceived Service Quality	0.160	0.069	0.137
Family Centeredness → Satisfaction	−0.010	0.541	0.919
Ongoing Care → Perceived Service Quality	0.081	0.192	0.385
Ongoing Care → Satisfaction	0.098	0.130	0.260
Perceived Service Quality → Revisit Intention	0.050	0.342	0.684
Perceived Service Quality → Satisfaction	0.059	0.248	0.496
Perceived Service Quality → Trust	−0.084	0.851	0.299
Satisfaction → Revisit Intention	0.062	0.356	0.713
Satisfaction → Trust	0.206	0.008	0.016
Trust → Revisit Intention	0.134	0.185	0.370
First Contact Utilisation → Perceived Service Quality	0.070	0.205	0.410
First Contact Utilisation → Satisfaction	−0.142	0.969	0.061

Source: Authors’ own work.
